# Regulation of LCoR and RIP140 expression in cervical intraepithelial neoplasia and correlation with CIN progression and dedifferentiation

**DOI:** 10.1007/s00432-020-03178-x

**Published:** 2020-03-10

**Authors:** Tilman L. R. Vogelsang, Elisa Schmoeckel, Christina Kuhn, Thomas Blankenstein, Mina Temelkov, Helene Heidegger, Theresa Maria Kolben, Thomas Kolben, Sven Mahner, Doris Mayr, Udo Jeschke, Aurelia Vattai

**Affiliations:** 1 Department of Obstetrics and Gynecology, University Hospital, LMU Munich, 80337 Munich, Germany; 2 Department of Obstetrics and Gynecology, University Hospital Augsburg, 86156 Augsburg, Germany; 3 Institute of Pathology, Faculty of Medicine, LMU Munich, 80337 Munich, Germany

**Keywords:** RIP140, LCoR, CIN, Cervical intraepithelial neoplasia, Cervical cancer

## Abstract

**Purpose:**

Ligand-dependent corepressor (LCoR) and receptor-interacting protein 140 (RIP140/NRIP1) play an important role in the regulation of multiple oncogenic signaling pathways and the development of cancer. LCoR and RIP140 form a nuclear complex in breast cancer cells and are of prognostic value in further prostate and cervical cancer. The purpose of this study was to analyze the regulation of these proteins in the development of cervical intraepithelial neoplasia (CIN I–III).

**Methods:**

Immunohistochemical analysis was obtained to quantify RIP140 and LCoR expression in formalin-fixed paraffin embedded tissue sections of cervical intraepithelial neoplasia samples. Tissue (*n* = 94) was collected from patients treated in the Department of Gynecology and Obstetrics, Ludwig-Maximilians-University of Munich, Germany, between 2002 and 2014. Correlations of expression levels with clinical outcome were carried out to assess for prognostic relevance in patients with CIN2 progression. Kruskal–Wallis test and Mann–Whitney *U* test were used for data analysis.

**Results:**

Nuclear LCoR overexpression correlates significantly with CIN II progression. Nuclear RIP140 expression significantly increases and nuclear LCoR expression decreases with higher grading of cervical intraepithelial neoplasia. Cytoplasmic RIP140 expression is significantly higher in CIN III than in CIN I or CIN II.

**Conclusion:**

A decrease of nuclear LCoR expression in line with an increase of dedifferentiation of CIN can be observed. Nuclear LCoR overexpression correlates with CIN II progression indicating a prognostic value of LCoR in cervical intraepithelial neoplasia. Nuclear and cytoplasmic RIP140 expression increases significantly with higher grading of cervical intraepithelial neoplasia underlining its potential role in the development of pre-cancerous lesions. These findings support the relevance of LCoR and RIP140 in the tumorigenesis indicating a possible role of LCoR and RIP140 as targets for novel therapeutic approaches in cervical intraepithelial neoplasia and cervical cancer.

## Introduction

Cervical cancer is the fourth most common cancer in females worldwide with more than 500,000 new cases each year (World Health Organization 2019, January 24). Furthermore, it is causing 7.5% of all cancer deaths in women (Ferlay et al. 2019). Due to routine cervical cancer screening methods such as HPV testing and cervical cytology (i.e. Pap smear test), the incidence of cervical cancer has decreased strongly, implicating the importance of the detection and treatment of pre-cancerous lesions, cervical intraepithelial neoplasia (CIN) (Schiffman and Wentzensen 2013). CINs are categorized into three grades (CIN I–III) depending on the amount of dysplastic epithelium involved. The major leading cause for the development of CIN and ultimately invasive cancer is a persistent infection with high-risk Human Papillomavirus (HR-HPV) (Schiffman et al. 2011). When expressed, the viral oncoprotein E6 disturbs the cell cycle by binding and degrading the tumor suppressor protein p53 (Gupta et al. 2003; Scheffner et al. 1990). The viral oncoprotein E7 disturbs the cell cycle by binding and degrading the retinoblastoma protein (pRb) and triggering E2F dissociation leading to proliferation of the cell and inhibition of cell death and differentiation (Chellappan et al. 1992; Wise-Draper and Wells 2008).

In the last 5–9 years, incidence of cervical intraepithelial neoplasia grades II and III has decreased by 30–50% due to HPV vaccination while incidence of CIN II and III has increased significantly by 19–23% in patients without HPV vaccination (Drolet et al. 2019).

Ligand dependent corepressor (LCoR) was initially described as a coregulator of estrogen receptor α (ERα) (Fernandes et al. 2003). Recent studies suggest its interaction with various transcription factors such as Krüppel-like factor 6 (KLF6) (Calderon et al. 2012) and peroxisome proliferator-activated receptor γ (PPARγ) (Shalom-Barak et al. 2018). It acts by recruiting histone deacetylases and C-terminal binding proteins (Palijan et al. 2009a, b). Asim and colleagues could show that LCoR inhibits prostate cancer growth in a xenograft mouse model via co-repression of activated androgen receptor (AR) (Asim et al. 2011).

Receptor-interacting protein of 140 kDa (RIP140), also known as nuclear receptor-interacting protein 1 (NRIP1), is described as a transcriptional coregulator of agonist-liganded ERα. Similar to LCoR, it functions by recruiting histone deacetylases and C-terminal binding proteins (Castet et al. 2004; Christian et al. 2004). RIP140 acts mostly as a co-repressor of multiple nuclear receptors and transcription factors and limits their transactivation (Augereau et al. 2006a, b; Cavailles et al. 1995).

RIP140 plays an important role in the progression and development of cancer (Aziz et al. 2015; Ghoussaini et al. 2012; Lapierre et al. 2014, 2015; Lei et al. 2015). In colon cancer, RIP140 is involved in Wnt-signaling and has a negative effect on Wnt/β-Catenin target genes and thereby inhibits cell proliferation, epithelial cell progression, and tumor growth (Lapierre et al. 2014, 2015). Direct interaction between RIP140 and E2F1 in breast cancer cell lines results in a repression of E2F1 target genes and could regulate cell proliferation (Docquier et al. 2010). Furthermore, RIP140 is essential for repressive activity of LCoR in breast cancer cell proliferation. LCoR overexpression and parallel downregulation of RIP140 mRNA leads to an increase in cell proliferation in breast cancer cell lines (Jalaguier et al. 2017). Low LCoR and RIP140 gene expression levels were associated with shorter overall survival (OS) in patients diagnosed with breast cancer (Jalaguier et al. 2017). Conversely, in a recent study we showed that RIP140 overexpression was associated with significant shorter overall survival of cervical cancer patients. RIP140 is not a significant negative prognosticator if LCoR expression is low (Vattai et al. 2017).

RIP140 and LCoR recruit similar cofactors implicated in transcriptional co-repression suggesting many parallels in their mechanism of action (White et al. 2004). Both RIP140 and LCoR bind to agonist-bound ligand binding domains (LBD), blocking coactivation in vivo (White et al. 2004). Multiple function and structure studies have displayed that RIP140 and LCoR recognize the same coactivator binding pockets of nuclear receptor LBDs (White et al. 2004).

Aim of this study was to analyze the expression of LCoR and RIP140 in cervical intraepithelial neoplasia grade I, II and III (CIN I–III) and the correlation of their expression regarding the progression of cervical dysplasia.

## Methods

Formalin-fixed paraffin embedded samples of 94 patients who had been treated at the Department of Gynecology and Obstetrics at Ludwig-Maximilians-University Munich, Germany, between 2002 and 2014 were included in this study. 81 slides could be obtained for analysis and 13 slides were not considered for analysis due to failed staining or no CIN staining on the slide. Patients were either diagnosed with CIN I (*n* = 38), CIN II (*n* = 26) or CIN III (*n* = 17). There has been no preselection of the patients. Histopathological grade of dysplasia and diagnosis were confirmed by a second gynecological pathologist. For progression analysis in CIN II samples, only patients with a follow-up visit and a histologically confirmed regress (*n* = 7) or progress (*n* = 17) were included. On their first visit all patients were tested positive for high risk Human Papillomavirus (Hybrid Capture 2, Quiagen). Initially, the tissue analyzed in this study had been collected due to routine histopathological diagnostics. All diagnostic procedures had been carried out beforehand.

### Immunohistochemistry

Immunohistochemical quantification of LCoR and RIP140 expression was obtained in the embedded samples of cervical dysplasia (CIN I–III). Immunohistochemical staining was obtained as described in earlier publications (Hester et al. 2019; Vattai et al. 2017). Tissue samples were surgically generated and instantly fixed in neutral buffered formalin (3.7%) followed by standardized paraffin bedding. Immunohistochemistry was initiated by deparaffinization of the formalin-fixed paraffin embedded tissue slices (3 µm) in xylol. Inactivation of endogenous peroxidase was obtained with 3% H_2_O_2_ in methanol for 20 min followed by a descending ethanol gradient for rehydration of the slides. Next, a pressure cooker filled with sodium citrate buffer (pH 6.0) was used to prepare the tissue for epitope retrieval. To prevent non-specific binding of the primary antibodies, blocking solution was applied. The tissue slides were incubated over night for 16 h consecutively with the following antibodies: anti-LCoR (polyclonal rabbit IgG, Novus Biologicals, Littleton, USA) and anti-RIP140 (polyclonal rabbit IgG, Sigma Aldrich, St. Louis, USA). Analyzation of the antibody reactivity was obtained with the ZytoChemPlus HRP Polymer System (mouse/rabbit) (Zytomed Systems, Berlin, Germany) according to the manufacturer’s protocol. Substrate and chromogen (3,3′-diaminobenzidine DAB; Dako, Glostrup, Denmark) was applied on the samples. Counterstaining was obtained with Mayer’s acidic hematoxylin. After dehydrating the slides in an ascending row of ethanol, the slides were cover slipped. Both nuclear and cytoplasmic staining of LCoR and RIP140 were further correlated with EP3 staining which has been carried out and published previously (Hester et al. 2019).

### Quantification

Analyzation of cervical dysplasia tissues was conducted by two different and independent observers using Leitz Diaplan microscope (Leitz, Wetzlar, Germany). To quantify each slide’s staining, the semiquantitative immunoreactive score (IRS) was used. Intensity and distribution pattern of the antigen are optically evaluated with the immunoreactive score (IRS) (Remmele and Stegner 1987). It was calculated by multiplying staining intensity (0: none; 1: weak; 2: moderate; 3: strong) with the number of positively stained cells (in %) (0: no staining, 1: < 10% of the cells; 2: 11–50%; 3: 51–80%; 4: > 80%). A scale from 0 (no expression) to 12 (very high expression) was used. Photos were taken with a CCD color camera (JVC, Victor Company of Japan, Japan).

### Statistical analysis

For data analysis IBM SPSS Statistics for Windows, Version 25, was used. *P *values *p* < 0.05 were considered statistically significant. Comparative analysis between different grades of CIN was obtained using nonparametric Kruskal–Wallis rank-sum test and Mann–Whitney *U* test. Spearman’s rank correlation coefficient was used for correlation analysis. Figures were designed using IBM SPSS Statistics for Windows, Version 25 as well as Microsoft® PowerPoint for Mac Version 16.30 (19101301).

## Results

### Nuclear LCoR expression in CIN grade I–III and correlation analysis with histopathological variables

**Fig. 1:**
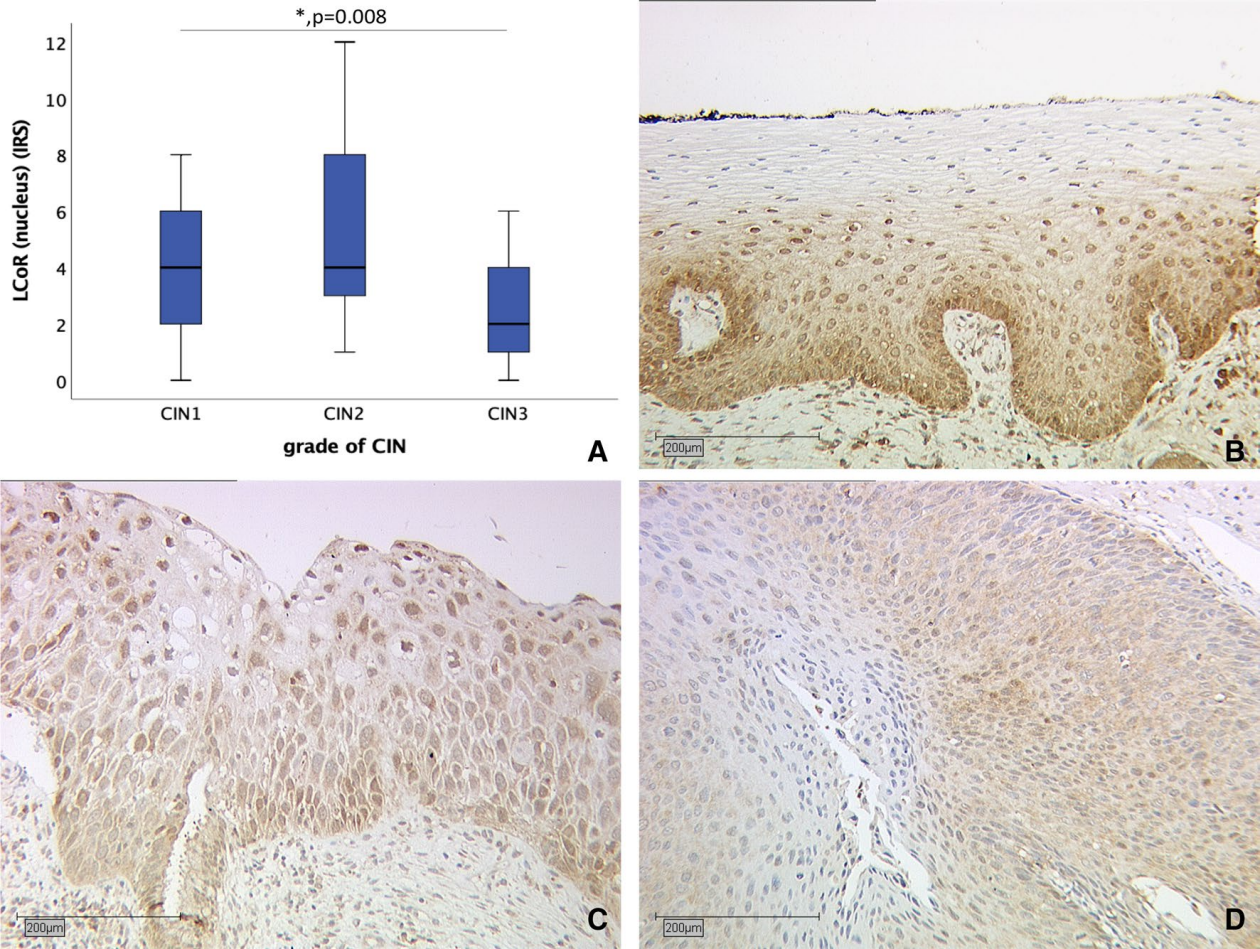
Correlation of nuclear LCoR expression (IRS) with grade of dysplasia. **a** Boxplot of nuclear LCoR expression and grade of dysplasia. **b** CIN I (*n* = 37) with nuclear LCoR IRS of 4; magnification × 10. **c** CIN II (*n* = 26) with nuclear LCoR IRS of 3; magnification × 10. **d** CIN III (*n* = 16) with nuclear LCoR IRS of 2; magnification × 10

Differences in nuclear LCoR expression were examined by comparing LCoR immunoreactive scores (IRS) between the groups of cervical tissue as shown in Fig. 1. While CIN I and CIN II showed a median IRS of four, median IRS in CIN III was two (*p* = 0.008). LCoR expression compared between CIN I and CIN II is not significantly changed (*p* = 0.088). Exemplary staining for all CIN grades is shown in Fig. 1.

For positive nuclear LCoR expression in cervical dysplasia tissue, a significant correlation with cytoplasmic LCoR (*p* = 0.014, Spearman Rho 0.270) was detected. Cytoplasmic RIP140 expression was negatively correlated with nuclear LCoR expression (*p* = 0.043; Spearman Rho − 0.224).

### RIP140 expression in CIN grade I–III

**Fig. 2:**
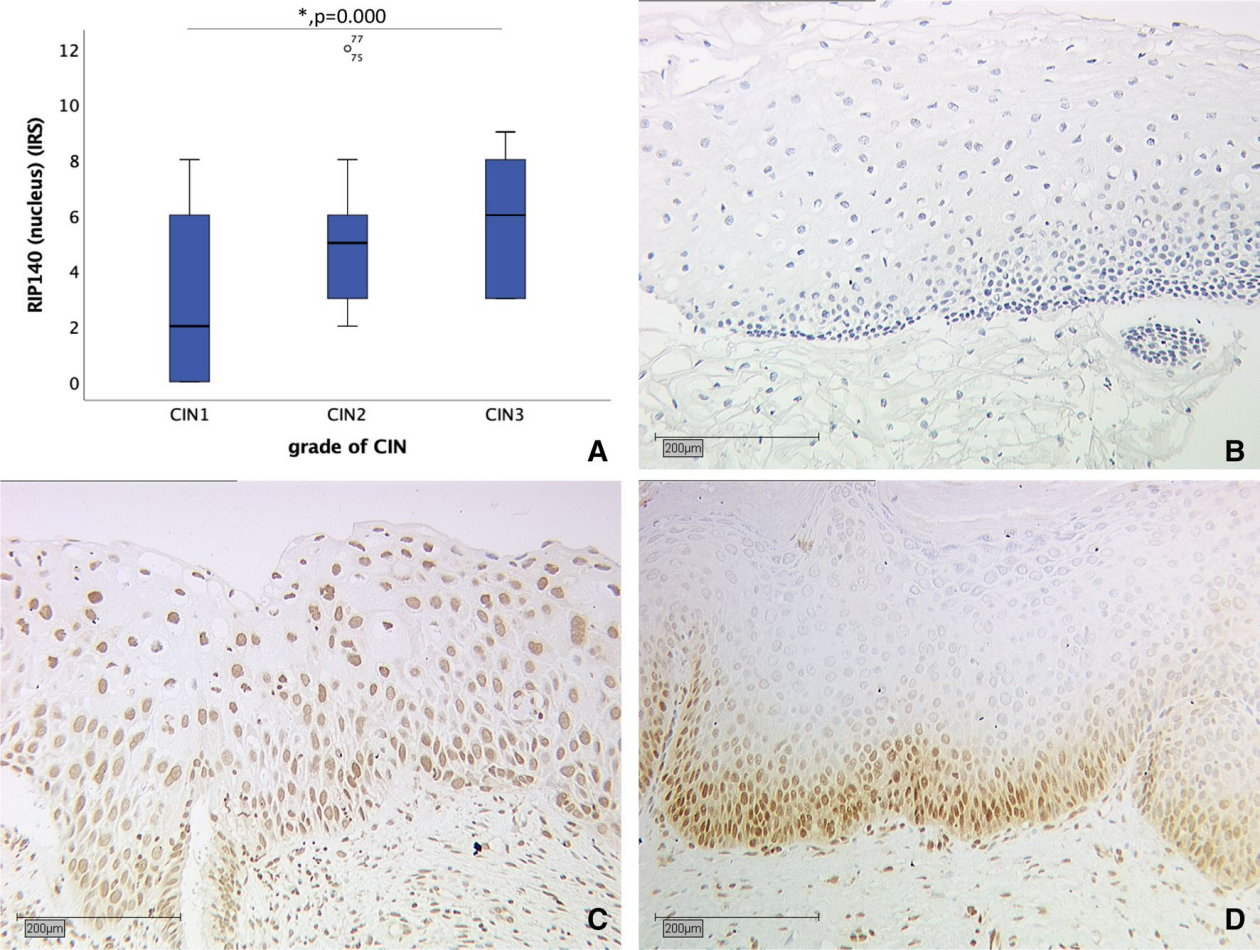
Correlation of nuclear RIP140 expression (IRS) with grade of dysplasia. **a** Boxplot of nuclear RIP140 expression and grade of dysplasia. **b** CIN I (*n* = 38) with nuclear RIP140 IRS of 1; magnification × 10. **c** CIN II (*n* = 26) with nuclear RIP140 IRS of six; magnification × 10. **d** CIN III (*n* = 17) with nuclear RIP140 IRS of six; magnification × 10

**Fig. 3:**
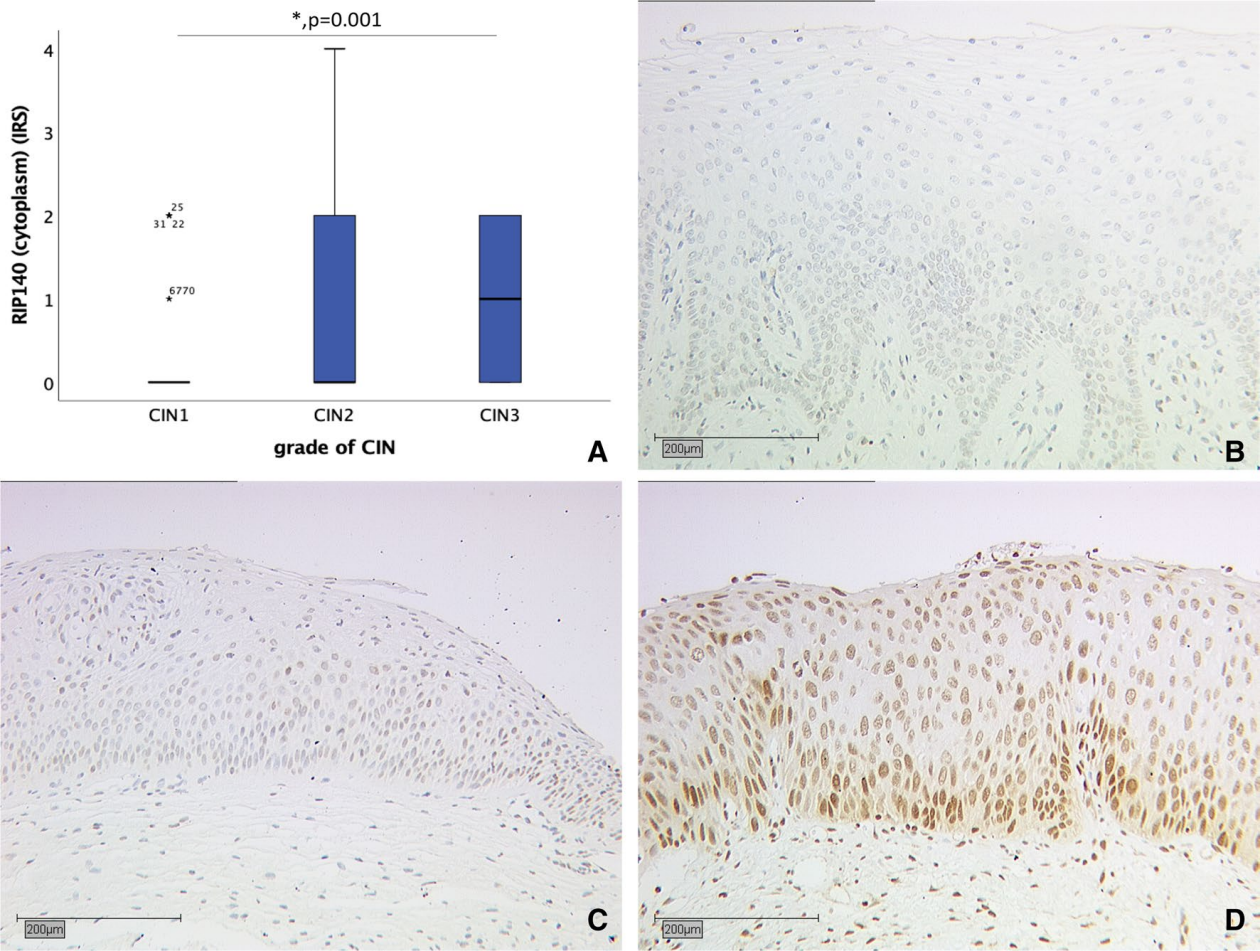
Correlation of cytoplasmic RIP140 expression (IRS) with grade of dysplasia. **a** Boxplot of cytoplasmic RIP140 expression and grade of dysplasia. **b** CIN I (*n* = 38) with cytoplasmic RIP140 IRS of 0; magnification × 25. **c** CIN II (*n* = 26) with cytoplasmic RIP140 IRS of zero; magnification × 10. **d** CIN III (*n* = 17) with cytoplasmic RIP140 IRS of two; magnification × 10

RIP140 expression was observed in the nucleus as well as the cytoplasm. In both compartments RIP140 expression significantly increased with higher grading of dysplasia as shown in Figs. 2 and 3. While CIN I showed a nuclear RIP140 expression with a median of two, the median in CIN II was five and in CIN III the median IRS was six (Kruskal–Wallis test *p* = 0.000). Cytoplasmic RIP140 expression in CIN I and CIN II with a median of zero increased significantly to the median of one in CIN III (Kruskal–Wallis test *p* = 0.001). Exemplary staining for all grades of CIN is shown in Figs. 2 and 3.

Correlation analysis showed that nuclear RIP140 expression correlated positively with cytoplasmic RIP140 (*p* = 0.000; Spearman Rho 0.552). Nuclear RIP140 correlated negatively with EP3 expression (*p* = 0.010; Spearman Rho − 0.290) in cervical dysplasia tissue. Cytoplasmic RIP140 expression correlated negatively with EP3 expression (*p* = 0.001, Spearman Rho − 0.365).

### Nuclear LCoR expression and progression of CIN

**Fig. 4:**
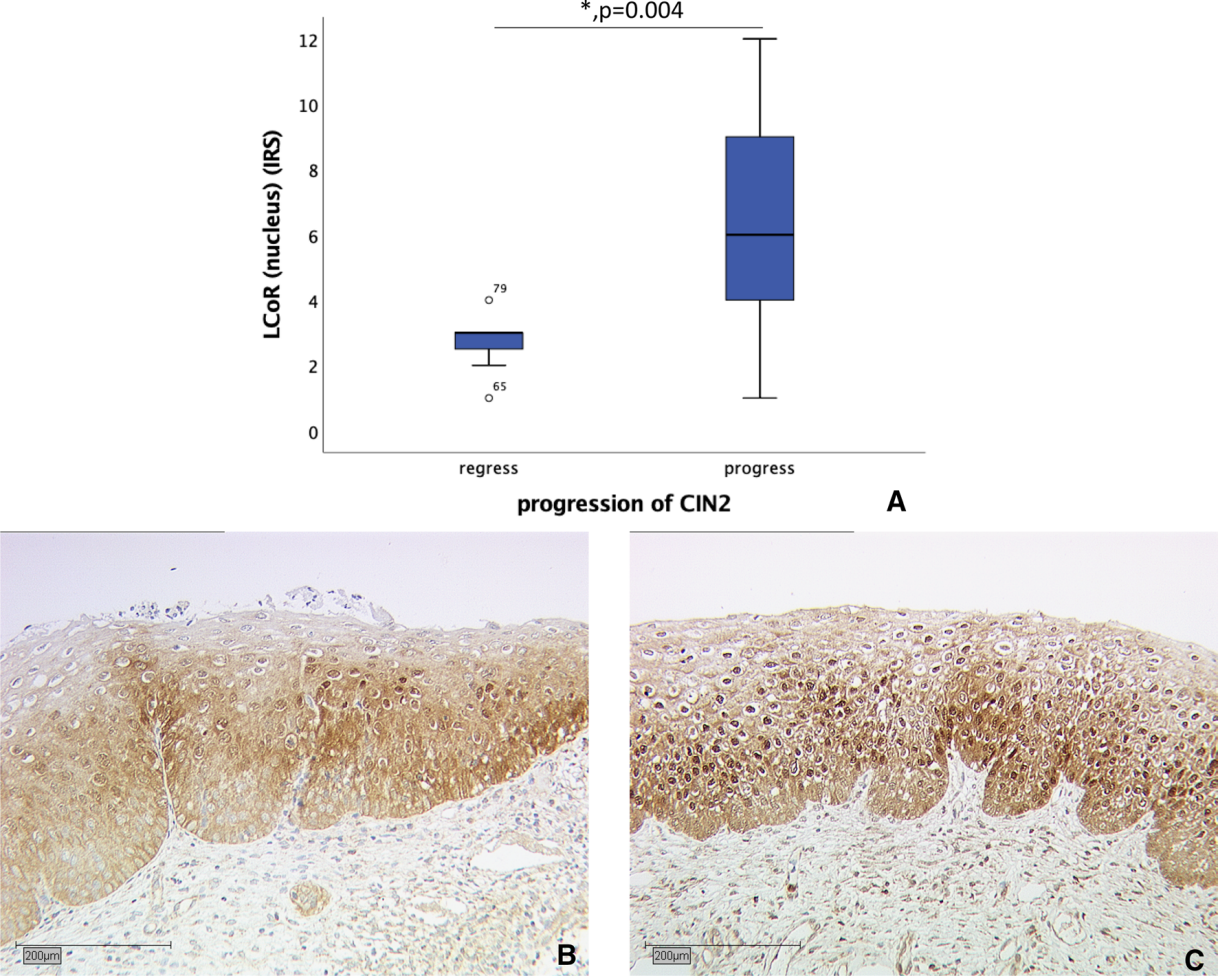
Correlation of nuclear LCoR expression (IRS) with CIN2 progression. **a** Boxplot of nuclear LCoR expression and CIN2 progression. **b** CIN II with regressive course (*n* = 7) with nuclear LCoR IRS of three; magnification × 10. **c** CIN II with progressive course (*n* = 17) with nuclear LCoR IRS of nine; magnification × 10

We compared nuclear LCoR expression between CIN II cases with histologically confirmed progress or regress to evaluate if LCoR expression is a prognostic marker for a progressive or regressive course in CIN. The median IRS of CIN II that showed a regressive course was three whereas the median IRS of CIN II with a progressive course was six (Fig. 4, Kruskal–Wallis test *p* = 0.004).

## Discussion

In a previous study we could show that patients with cervical cancer expressing low levels of LCoR and RIP140 correlate with a better overall survival than patients expressing high levels of RIP140 (Vattai et al. 2017). RIP140 is an independent predictor of poor OS in patients with cervical cancer (Vattai et al. 2017). In the current study we could show that nuclear RIP140 expression increases significantly with the cervical dysplasia grade. In line with our findings, RIP140 plays a role in different molecular pathways that affect the development of cervical cancer such as the estrogen receptor signaling (Lapierre et al. 2015). Elevated estrogen levels lead to a higher risk of cervical intraepithelial neoplasia as well as cervical cancer in HPV-infected patients (Ramachandran 2017).

Besides its influence on estrogen receptor signaling, RIP140 represses transactivation of E2F1 and inhibits expression of several E2F1 target genes in breast cancer cell lines (Docquier et al. 2010). E2F1 is a transcriptional activator that plays an essential role in the regulation of cell proliferation, apoptosis, G1/S transition and S-phase entry during the cell cycle (Chen et al. 2009; Dimova and Dyson 2005). It can bind to and is regulated by the tumor suppressor protein retinoblastoma (pRb) (McNair et al. 2018). Phosphorylation of pRb by G2-M and S-phase cyclin dependent kinases releases E2F1 and allows it to transcribe its target genes resulting in cell cycle progression (Weinberg 1995). The degradation of E2F repressor pRb by the HPV oncoprotein E7 via the ubiquitin–proteasome pathway results in activation of E2F-regulated genes and consequently deregulates the progression through the G1 phase of the cell cycle (Boyer et al. 1996; Rosty et al. 2005). In cervical cancer, E2F1 expression is significantly increased suggesting that genes which are involved in invasive cervical carcinoma are regulated by E2F (Rosty et al. 2005; Srivastava et al. 2014).

Another pathway influenced by RIP140 is Wnt/β-catenin signaling which is involved in cancer progression. Lapierre et al. (2014) showed a suppressive effect of RIP140 on Wnt/β-catenin target genes in colon cancer. This stands in contrast to the previously described role of RIP140 in cervical and breast cancer and to our results in CIN indicating the complexity of RIP140 regulation (Aziz et al. 2015; Vattai et al. 2017). The Wnt/β-catenin signaling pathway has been described in HPV-related tumors implicating potential mechanisms by which the viral oncoproteins E6 and E7 activate this pathway (Bello et al. 2015).

In CIN III, cytoplasmic RIP140 expression is significantly higher than in CIN I or CIN II. Nucleo-cytoplasmic shuttling or a higher transcription followed by modification of RIP140 might explain the cytoplasmic increase. After transcription of genes in the nucleus, proteins are transported to the cytoplasm for translation and modification (Fu et al. 2018). For shuttling, nuclear pore complexes (NPCs) selectively transport cargoes across the nuclear envelope (Alber et al. 2007). Nucleo-cytoplasmic shuttling plays an important role in activity of proteins, signaling pathways, and thereby tumorigenesis (Shreberk-Shaked and Oren 2019). Post-translational modifications such as lysine acetylation (Vo et al. 2001) or conjugation to Vitamin-B6 (Huq et al. 2007) might play a role in nucleo-cytoplasmic shuttling.

LCoR is described as a tumor suppressor in prostate cancer and an inhibitor of cell growth in prostatic cancer cells (Asim et al. 2011). In breast cancer cell lines, LCoR is regulated by RIP140 and inhibits cell proliferation. Jalaguier and colleagues (2017) could show that LCoR mRNA is expressed higher in breast cancer cell lines than in normal samples. In this study, we could show that high nuclear LCoR expression correlates significantly with CIN II progression. High LCoR expression might thereby lead to a higher grade of dysplasia and towards tumorigenesis. Interestingly, high LCoR expression furthermore correlates significantly with low dysplasia grade. In general, 50% of histologically confirmed CIN II lesions show a regressive course while only 18% progress to CIN III or worse within 2 years of surveillance (Tainio et al. 2018). Cervical dysplasia is common in young women and it has been controversially discussed whether or not CIN II is an indication for surgical treatment since loop electrosurgical excision procedure (LEEP) is associated with a significant higher risk of premature birth in following pregnancies (Frega et al. 2018). Therefore, it is of high importance to differentiate between a potentially progressive and regressive CIN II.

Correlations of LCoR and RIP140 expression have been described in studies on breast, cervical, and gastrointestinal cancer (Jalaguier et al. 2017; Triki et al. 2017; Vattai et al. 2017). In our study, we detected a negative correlation between nuclear LCoR and cytoplasmic RIP140 expression (*p* = 0.005). Correlation of nuclear RIP140 and nuclear LCoR expression was not significant.

In conclusion, in our hypothesis generating study we observed that RIP140 as well as LCoR are expressed differently in all grades of cervical intraepithelial neoplasia, with the exception of LCoR expression compared between CIN I and CIN II, suggesting that LCoR and RIP140 play a relevant role in carcinogenesis of cervical cancer. Additionally, LCoR expression appears to be a marker for CIN II progression. Further experiments are required to analyze whether LCoR can be considered as an additional diagnostic factor to help in the decision-making process regarding non-surgical treatment eligibility of CIN II patients.

## Data Availability

The datasets generated and analyzed during the current study are available from the correspondent author on reasonable request.

## Abbreviations

CIN: Cervical intraepithelial neoplasia
LCoR: Ligand-dependent corepressor
RIP140: Receptor-interacting protein of 140 kDa
NRIP1: Nuclear receptor-interacting protein 1 (= RIP140)
Erα: Estrogen receptor *α*
KLF6: Krüppel-like factor 6
pRb: Retinoblastoma protein
OS: Overall survival
